# Editorial: Developmental therapeutics in pediatric neuro-oncology

**DOI:** 10.3389/fonc.2023.1134168

**Published:** 2023-01-24

**Authors:** Atsushi Makimoto, Anat Erdreich-Epstein, Yoshihiro Muragaki

**Affiliations:** ^1^ Department of Laboratory Medicine, Tokyo Metropolitan Children’s Medical Center, Tokyo, Japan; ^2^ Department of Hematology/Oncology, Tokyo Metropolitan Children’s Medical Center, Tokyo, Japan; ^3^ Cancer and Blood Disease Institute, Department of Pediatrics, Children’s Hospital Los Angeles and Norris Comprehensive Cancer Center, Keck School of Medicine, University of Southern California, Los Angeles, CA, United States; ^4^ Department of Pathology, Children’s Hospital Los Angeles and Norris Comprehensive Cancer Center, Keck School of Medicine, University of Southern California, Los Angeles, CA, United States; ^5^ Institute of Advanced Biomedical Engineering and Science, Tokyo Women’s Medical University, Tokyo, Japan; ^6^ Center for Advanced Medical Engineering Research and Development, Kobe University, Kobe, Japan

**Keywords:** central nervous system (CNS) tumors, children and adolescent, developmental therapeutics, clinical trials, risk-based approach, cancer genetics, molecular biology

Central nervous system (CNS) tumors are the most common form of solid tumors in children and adolescents between the age of 0 years and 19 years. These tumors comprise a variety of disease entities including tumors of glial origin, embryonal origin, germ cell origin, and others. While the annual incidence of brain tumors in pediatric patients (6.14 per 100,000 population) is lower than in adults (23.79 per 100,000), they are the most common cause of cancer–related mortality in children (1). Multidisciplinary treatment strategies consisting of surgery, radiotherapy, and/or chemotherapy are used in effort to control these tumors and are commonly associated with acute and chronic adverse events. The topic of this special issue in *Frontiers in Oncology*, “Developmental Therapeutics in Pediatric Neuro-oncology”, comes to shed light on emerging treatment strategies intended to improve outcome of children and adolescents with CNS tumors and to minimize currently-associated toxicities and complications related to therapy.

A molecularly-informed subclassification of pediatric CNS tumors in the latest WHO classification (2) continues a paradigm shift that is altering the traditional morphological and risk-based approach to pediatric brain tumors and generating new opportunities for novel therapies, including molecularly targeted ones. One of the earliest subclassifications was that of medulloblastoma (MB), the most common malignant CNS tumor in children. Based on molecular profiling the early classification designated four molecularly-distinct MB subgroups: Wingless (WNT), Sonic Hedgehog (SHH), Group 3, and Group 4 (3, 4). Shifts to molecular classification are also prominent in ependymomas, gliomas, and other CNS tumors. These classifications continue to be further refined based on newly-discovered molecular features (5, 6). Although molecular diagnostics are rapidly advancing our understanding of pediatric brain tumors, shifts in treatment take longer to catch up and many of the emerging therapeutic approaches are still in the realm of clinical trials.

This Research Topic of *Frontiers in Oncology* includes three review articles discussing novel treatment strategies based on recent progress in molecular/genetic profiling. McSwain et al. describe the potential for a variety of proteins responsible for DNA damage repair as a part of targeted therapy applicable to MB of all molecular subtypes. They discuss molecular underpinnings of genomic instability in MB and potential avenues for its exploitation through inhibition of the DNA damage response. Schwark et al. discuss the application of receptor tyrosine kinase (RTK) targeting in pediatric high-grade gliomas and diffuse midline gliomas, two of the deadliest forms of cancer in children and adolescents. They review recent advances in murine modeling and precision targeting of the most important RTKs in the clinical context. Obviously, not all available mouse models could be included, and another that deserves mention is the versatile and elegant Mosaic Analysis by Dual Recombinase-mediated cassette exchange (MADR) mouse model that has already generated autochthonous H3K27M and H3G34R high grade gliomas and several subtypes of ependymomas (7). Finally, Takami and Ichimura present a comprehensive review of biomarkers in risk-based treatment modifications for CNS germ cell tumors (GCTs). Their profound understanding of the biology and pathogenesis of GCTs will hopefully contribute to improving treatment outcomes and to reducing long-term treatment-related adverse effects through better treatment stratification that is based on precise assessment of the treatment failure risk.

In addition to these review articles, this issue includes three original research articles discussing novel treatment modalities applicable to CNS tumors in children and adolescents. Goldman et al. present safety data on tumor treating fields (TTFields) therapy in pediatric patients using post-marketing surveillance data. While TTFields therapy is now part of the standard of care for adult glioblastoma (8), pediatric clinical trials have only recently begun (Goldman et al., 9). Chiba et al. discuss the results of their Phase I/II clinical trial of photodynamic therapy (PDT) for malignant CNS tumors in children and adolescents. Although the results confirmed the safety of PDT in nine procedures in eight patients, future late-phase trials are needed to confirm its efficacy. Mizumoto et al. reported on light flash and odor in children during proton-beam therapy, which is increasingly being used for the treatment of pediatric cancers. Unexpected adverse events associated with novel treatment modalities are often very difficult to predict. In this regard, not only clinical trials, but also methodologies such as prospective trials and observational studies, should be reappraised.

The brief overview presented here of the articles in this issue demonstrates that persistent effort of numerous experts and creative application of the most recent research findings continue to contribute greatly to advancing pediatric neuro-oncology and to improving clinical outcomes in children and adolescents with CNS tumors. Due to space limitations, some active fields in development in pediatric neuro-oncology such as immunotherapy were left for future issues.

The editors would like to express many thanks to the authors, reviewers, and members of the editorial board for helping make this special issue of *Frontiers in Oncology* a success. We hope that these six articles will be of interest to readers and inspire them to continue the joint effort to develop more effective and less toxic treatments for CNS tumors in children and adolescents.

## Author contributions

All authors made substantial, intellectual contribution to the work, edited the manuscript, and approved the its submission for publication.
